# Supplementary material for: Meta-Analysis and Gene Set Enrichment Relative to ER Status Reveal Elevated Activity of MYC and E2F in the “Basal” Breast Cancer Subgroup
Source: PLoS One. 2009 Mar 9;4(3):e4710. doi: 10.1371/journal.pone.0004710 (PMC2650420; doi:10.1371/journal.pone.0004710)
Supplement: Dataset S1 — GSEA results from first screen. The file contains a zipped archive of the HTML results of the second GSEA screen. Reports for enriched or depleted datasets may be accessed from the “index.html” link within the my_analysis.GseaPreranked.1219975950625.rpt.zip ZIP archive. Enrichment plots are available for each of the 50 most enriched and depleted gene sets. (13.32 MB ZIP) [file pone.0004710.s001.zip › my_analysis.GseaPreranked.1219975950625.rpt.zip!//CHR4P16.html]

Details for gene set CHR4P16[GSEA]

|  || Dataset | zSco.ERneg.to.ERpos\_collapsed |
| Phenotype | NoPhenotypeAvailable |
| Upregulated in class | na\_neg |
| GeneSet | CHR4P16 |
| Enrichment Score (ES) | -0.40330145 |
| Normalized Enrichment Score (NES) | -1.7315022 |
| Nominal p-value | 0.003236246 |
| FDR q-value | 0.0527563 |
| FWER p-Value | 0.935 |
Table: GSEA Results Summary

  

Fig 1: Enrichment plot: CHR4P16      
 Profile of the Running ES Score & Positions of GeneSet Members on the Rank Ordered List

  

| PROBE | GENE SYMBOL | GENE\_TITLE | RANK IN GENE LIST | RANK METRIC SCORE | RUNNING ES | CORE ENRICHMENT || 1 | TACC3 |  |  | 669 | 3.995 | -0.0176 | No |
| 2 | TLR6 |  |  | 990 | 3.496 | -0.0130 | No |
| 3 | SH3BP2 |  |  | 1136 | 3.323 | 0.0035 | No |
| 4 | HS3ST1 |  |  | 1563 | 2.891 | -0.0049 | No |
| 5 | D4S234E |  |  | 1832 | 2.669 | -0.0032 | No |
| 6 | SOD3 |  |  | 2135 | 2.429 | -0.0059 | No |
| 7 | C4ORF9 |  |  | 2378 | 2.271 | -0.0055 | No |
| 8 | SLBP |  |  | 2921 | 1.974 | -0.0302 | No |
| 9 | HMX1 |  |  | 3414 | 1.742 | -0.0529 | No |
| 10 | KLF3 |  |  | 3927 | 1.544 | -0.0789 | No |
| 11 | C4ORF23 |  |  | 4469 | 1.331 | -0.1087 | No |
| 12 | SLC2A9 |  |  | 4684 | 1.258 | -0.1145 | No |
| 13 | WHSC1 |  |  | 4717 | 1.247 | -0.1067 | No |
| 14 | SH3TC1 |  |  | 4735 | 1.241 | -0.0978 | No |
| 15 | MED28 |  |  | 4981 | 1.164 | -0.1067 | No |
| 16 | GRPEL1 |  |  | 5063 | 1.130 | -0.1035 | No |
| 17 | DRD5 |  |  | 5130 | 1.107 | -0.0993 | No |
| 18 | C4ORF6 |  |  | 5518 | 0.968 | -0.1206 | No |
| 19 | IDUA |  |  | 6130 | 0.775 | -0.1603 | No |
| 20 | AFAP |  |  | 6183 | 0.755 | -0.1580 | No |
| 21 | EVC |  |  | 6224 | 0.739 | -0.1549 | No |
| 22 | LETM1 |  |  | 6363 | 0.697 | -0.1596 | No |
| 23 | C4ORF10 |  |  | 6562 | 0.630 | -0.1694 | No |
| 24 | S100P |  |  | 6721 | 0.580 | -0.1765 | No |
| 25 | RGS12 |  |  | 7051 | 0.469 | -0.1975 | No |
| 26 | WDR1 |  |  | 7353 | 0.366 | -0.2172 | No |
| 27 | LOC152719 |  |  | 7623 | 0.261 | -0.2353 | No |
| 28 | SLC26A1 |  |  | 8258 | 0.013 | -0.2830 | No |
| 29 | ABLIM2 |  |  | 8407 | -0.040 | -0.2939 | No |
| 30 | HGFAC |  |  | 8493 | -0.074 | -0.2997 | No |
| 31 | CRMP1 |  |  | 8558 | -0.105 | -0.3036 | No |
| 32 | TNIP2 |  |  | 8613 | -0.134 | -0.3066 | No |
| 33 | MFSD7 |  |  | 9578 | -0.606 | -0.3743 | No |
| 34 | CYTL1 |  |  | 9618 | -0.628 | -0.3721 | No |
| 35 | MXD4 |  |  | 9642 | -0.639 | -0.3686 | No |
| 36 | WHSC2 |  |  | 9680 | -0.659 | -0.3660 | No |
| 37 | MSX1 |  |  | 9893 | -0.779 | -0.3756 | No |
| 38 | DGKQ |  |  | 9950 | -0.811 | -0.3731 | No |
| 39 | ABCA11 |  |  | 10031 | -0.863 | -0.3720 | No |
| 40 | BAPX1 |  |  | 10154 | -0.932 | -0.3736 | No |
| 41 | SPON2 |  |  | 10204 | -0.961 | -0.3693 | No |
| 42 | FGFR3 |  |  | 10244 | -0.985 | -0.3642 | No |
| 43 | CTBP1 |  |  | 10463 | -1.130 | -0.3713 | No |
| 44 | RAB28 |  |  | 10517 | -1.176 | -0.3657 | No |
| 45 | ZNF141 |  |  | 10679 | -1.304 | -0.3671 | No |
| 46 | ADRA2C |  |  | 10807 | -1.391 | -0.3652 | No |
| 47 | C4ORF8 |  |  | 11313 | -1.835 | -0.3882 | Yes |
| 48 | TETRAN |  |  | 11410 | -1.943 | -0.3794 | Yes |
| 49 | GRK4 |  |  | 11513 | -2.049 | -0.3703 | Yes |
| 50 | ADD1 |  |  | 11683 | -2.204 | -0.3649 | Yes |
| 51 | CPZ |  |  | 11799 | -2.326 | -0.3544 | Yes |
| 52 | HD |  |  | 11959 | -2.513 | -0.3457 | Yes |
| 53 | MAEA |  |  | 11975 | -2.533 | -0.3260 | Yes |
| 54 | PDE6B |  |  | 12010 | -2.573 | -0.3074 | Yes |
| 55 | PCGF3 |  |  | 12110 | -2.716 | -0.2925 | Yes |
| 56 | PIGG |  |  | 12252 | -2.912 | -0.2792 | Yes |
| 57 | C4ORF15 |  |  | 12271 | -2.930 | -0.2564 | Yes |
| 58 | MAN2B2 |  |  | 12294 | -2.962 | -0.2337 | Yes |
| 59 | RNF4 |  |  | 12351 | -3.025 | -0.2130 | Yes |
| 60 | LDB2 |  |  | 12491 | -3.251 | -0.1968 | Yes |
| 61 | MRFAP1L1 |  |  | 12548 | -3.335 | -0.1735 | Yes |
| 62 | LRPAP1 |  |  | 12709 | -3.635 | -0.1557 | Yes |
| 63 | GAK |  |  | 12742 | -3.723 | -0.1275 | Yes |
| 64 | STK32B |  |  | 13030 | -4.556 | -0.1116 | Yes |
| 65 | KIAA0232 |  |  | 13171 | -5.183 | -0.0795 | Yes |
| 66 | WFS1 |  |  | 13210 | -5.457 | -0.0375 | Yes |
| 67 | STX18 |  |  | 13216 | -5.550 | 0.0078 | Yes |
Table: GSEA details [plain text format]

  

Fig 2: CHR4P16: Random ES distribution      
 Gene set null distribution of ES for **CHR4P16**
